# Mast cells enhance sterile inflammation in chronic nonbacterial osteomyelitis

**DOI:** 10.1242/dmm.040097

**Published:** 2019-08-20

**Authors:** Stephanie Young, Namit Sharma, Jae Hoon Lee, Violeta Chitu, Volker Neumeister, Elisabeth Sohr, E. Richard Stanley, Christian M. Hedrich, Andrew W. B. Craig

**Affiliations:** 1 Department of Biomedical and Molecular Sciences, Queen's University, Kingston, ON K7L 3N6, Canada; 2 Department of Developmental and Molecular Biology, Albert Einstein College of Medicine, Bronx, NY 10461, USA; 3 Departments of Clinical Chemistry and Laboratory Medicine, University Hospital Carl Gustav Carus, Technical University Dresden, Dresden 01307, Germany; 4 Pediatric Rheumatology and Immunology, Children's Hospital Dresden, Technical University Dresden, Dresden 01307, Germany; 5 Department of Women's & Children's Health, Institute of Translational Medicine, University of Liverpool, Liverpool L14 5AB, UK; 6 Department of Paediatric Rheumatology, Alder Hey Children's NHS Foundation Trust Hospital, Liverpool L14 5AB, UK

**Keywords:** Autoinflammation, Chronic recurrent multifocal osteomyelitis, Cytokines, Interleukin-1β, Bone disease, CNO

## Abstract

Chronic nonbacterial osteomyelitis (CNO) is an autoinflammatory bone disease, and patients with active or recurrent bone inflammation at multiple sites are diagnosed with chronic recurrent multifocal osteomyelitis (CRMO). The Chronic multifocal osteomyelitis (CMO) mouse model develops IL-1β-driven sterile bone lesions reminiscent of severe CRMO. The goal of this study was to evaluate the potential involvement of mast cells in CMO/CRMO. Here, we show that mast cells accumulate in inflamed tissues from CMO mice and that mast cell protease Mcpt1 can be detected in the peripheral blood. A transgenic model of connective tissue mast cell depletion (*Mcpt5-Cre:Rosa26-Stop*^fl/fl^*-DTa*) was crossed with CMO mice and the resulting mice (referred to as CMO/MC^–^) showed a significant delay in disease onset compared with age-matched CMO mice. At 5-6 months of age, CMO/MC^–^ mice had fewer bone lesions and immune infiltration in the popliteal lymph nodes that drain the affected tissues. In bone marrow-derived mast cell cultures from CMO mice, cytokine production in response to the alarmin IL-33 was elevated compared with wild-type cultures. To test the relevance of mast cells to human CRMO, we tested serum samples from a cohort of healthy controls and from CRMO patients at diagnosis. Interestingly, mast cell chymase was elevated in CRMO patients as well as in patients with oligoarticular juvenile arthritis. Tryptase-positive mast cells were also detected in bone lesions from CRMO patients and patients with bacterial osteomyelitis. Together, our results identify mast cells as cellular contributors to bone inflammation in CMO/CRMO and provide rationale for further study of mast cells as therapeutic targets.

## Abstract (precis)

**Summary:** This paper reports that mast cells promote bone loss in an autoinflammatory disease model and that mast cell mediators were detected in autoinflammatory disease patient samples.

## INTRODUCTION

Chronic nonbacterial osteomyelitis (CNO) is an autoinflammatory bone disease (Zhao and Ferguson, 2018). Although some patients exhibit bone lesions at single sites, most patients develop chronically active or recurrent bone inflammation at multiple sites, and are then diagnosed with recurrent multifocal osteomyelitis (CRMO) (Ferguson et al., 2006; Ferguson and Laxer, 2015; Ferguson and Sandu, 2012; Hofmann et al., 2016b; Schnabel et al., 2016). CRMO partially resembles other syndromic autoinflammatory bone diseases, such as deficiency of the interleukin (IL)-1 receptor antagonist (DIRA) or Majeed syndrome (Aksentijevich et al., 2009; Ferguson et al., 2005). CRMO patients with active disease are characterized by elevated pro-inflammatory cytokines [tumor necrosis factor (TNF)-α, IL-1β, IL-6 and IL-8] in the serum (Hofmann et al., 2016a), and reduced IL-10 production in peripheral blood monocytes (Hofmann et al., 2011). In CRMO patient samples, reduced IL-10 expression correlates with increased inflammasome expression and activation (Brandt et al., 2018), leading to increased IL-1β-driven inflammatory bone loss (Cox and Ferguson, 2018; Hofmann et al., 2015; Zhao and Ferguson, 2018).

Several key features of severe CRMO are modeled in chronic multifocal osteomyelitis (CMO) mice (Chitu et al., 2009; Ferguson et al., 2006). CMO mice carry destabilizing homozygous L98P mutations in the gene encoding proline-serine-threonine phosphatase-interacting protein 2 (*Pstpip2*) (Chitu et al., 2009; Ferguson et al., 2006). Expression of PSTPIP2 protein is restricted to hematopoietic progenitors and innate immune cells (Cassel et al., 2014; Chitu et al., 2009; Liu et al., 2014; Lukens et al., 2014a). PSTPIP2 suppresses differentiation and the effector function of macrophages (Chitu et al., 2009, 2005; Tsujita et al., 2013), neutrophils (Cassel et al., 2014; Lukens et al., 2014a,b; Netea et al., 2015) and osteoclasts (Chitu et al., 2012). This could be explained by PSTPIP2 promoting plasma membrane recruitment of negative regulators of immune cell activation (Itoh et al., 2005; Tsujita et al., 2006), including PEST protein-tyrosine phosphatases (Chitu et al., 2012; Drobek et al., 2015; Wu et al., 1988), C-terminal Src kinase (Csk) (Drobek et al., 2015) and the phosphatidylinositol-3,4,5-trisphosphate 5-phosphatase SHIP1 (Drobek et al., 2015). These interactions are enhanced in response to immune cell activation by a variety of receptors (Chitu et al., 2005; Drobek et al., 2015; Yeung et al., 1998). PSTPIP2-deficient neutrophils show elevated responses to inflammatory stimuli (lipopolysaccharide, FcR capping, crystalline silica) (Cassel et al., 2014; Drobek et al., 2015), probably caused by increased Src/Ras/Erk (Cloutier and Veillette, 1996, 1999) and by PI3K/Akt signaling (Huber and Gibbs, 2015; Rauh et al., 2004). However, PSTPIP2 is also expressed in other cells, including mast cells (Chitu et al., 2009), but no functional studies have been reported to date.

Mast cells are specialized innate immune cells residing near potential portals of entry for pathogens (Tsai et al., 2011). Mast cell degranulation results in the release of mast cell proteases (chymase, tryptase), cytokines/chemokines (TNF-α, CXCL1/KC) and histamine that all promote tissue inflammation, leukocyte recruitment and blood vessel permeability (De Filippo et al., 2013; Wernersson and Pejler, 2014). Mast cell activation also leads to rapid production and release of lipid mediators implicated in leukocyte recruitment and tissue inflammation (Haeggström and Funk, 2011; Satpathy et al., 2015). Studies using transgenic models of connective tissue mast cell (CTMC) ablation implicated key roles of mast cells in allergic skin inflammation and arthritis (Dudeck et al., 2011, 2015; Schubert et al., 2015). Hence, mast cells and their mediators could contribute to inflammatory disorders driven by aberrantly activated innate immune cells, such as found in CMO and CRMO.

In this study, we demonstrate that mast cells accumulate in inflammatory bone lesions in CMO mice and that genetic ablation of CTMCs results in delayed onset and reduced severity of the inflammatory disease. Elevated inflammatory cytokine production in bone marrow-derived mast cells from CMO mice in response to IL-33 suggests that mast cells are hyperactive in the CMO model. Furthermore, we detected mast cell infiltrates in bone tissue lesions of CRMO patients and the release of mast cell chymase in the serum of CRMO patients at diagnosis.

## RESULTS

### Evidence of CTMC activation and role in chronic multifocal osteomyelitis

**Fig. 1.**
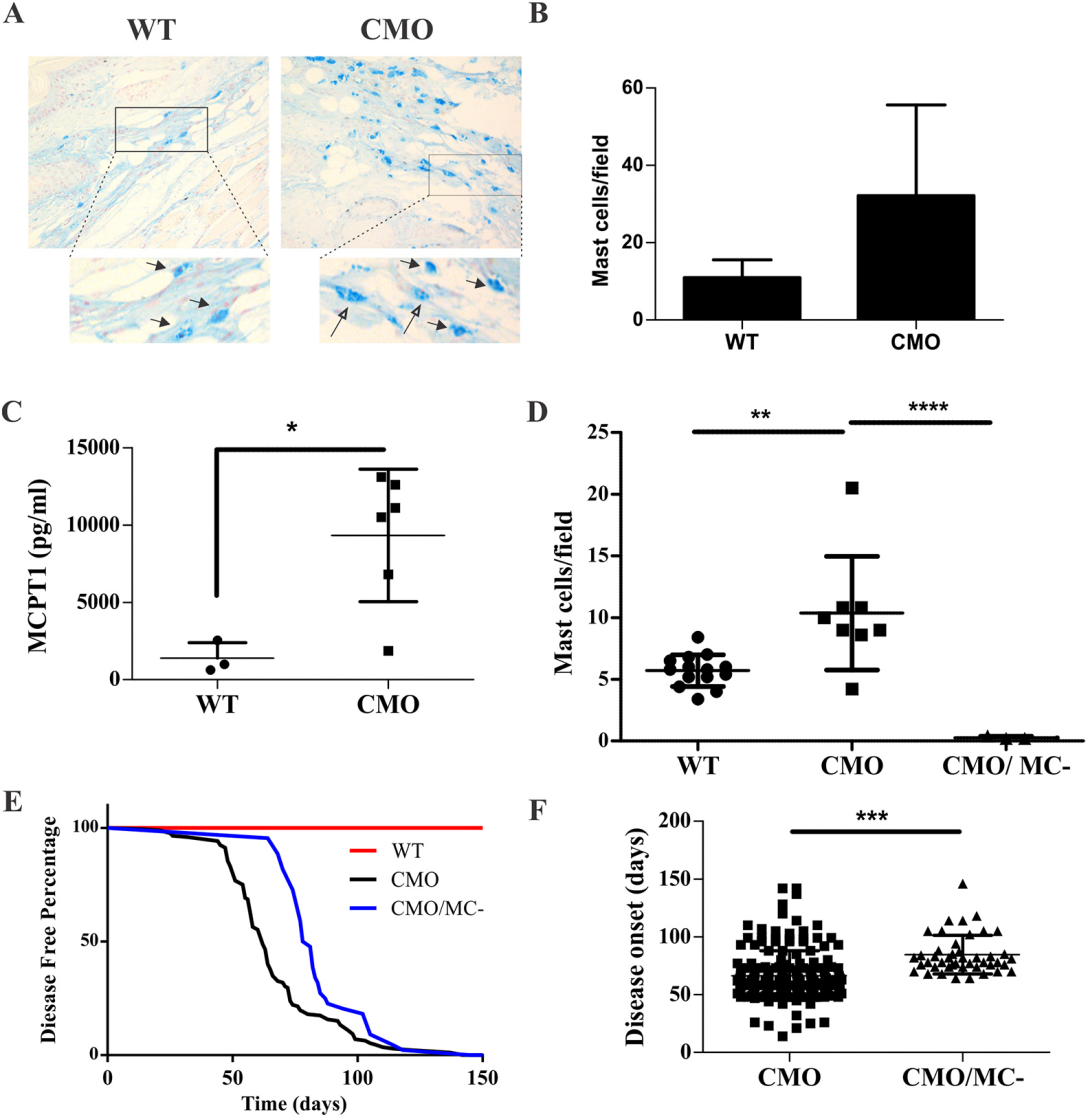
**CTMCs promote CMO disease onset.** (A) Representative images of mast cells detected with Alcian Blue staining of decalcified tail tissue sections from 5-month-old WT and CMO male mice (insets show magnified view with arrows indicating mast cells). (B) Quantification of mast cell density in WT and CMO tail tissue sections stained with Alcian Blue. Graph depicts the average mast cell density per field (mean±s.d., 5 fields/sample, 3 mice/group). (C) Levels of MCPT1 were profiled in plasma from 5-month-old male WT (*n*=3) and CMO (*n*=6) mice by ELISA. (D) Quantification of mast cell populations in Alcian Blue-stained ear tissue sections (*n*=6 per group, 5 fields/mouse). (E) Kaplan–Meier disease-free survival curve is shown for WT (*n*=50), CMO (*n*=172), and CMO/MC^–^ (*n*=42) mice. (F) Average age of disease onset in CMO (*n*=40) and CMO/MC^–^ (*n*=43) mice. *P<0.05, **P<0.01, ***P<0.001, ***P<0.001.

To test the relevance of CTMCs to CMO disease pathophysiology, we collected tail tissues from 5-month-old wild-type (WT) and *Pstpip2*^cmo/cmo^ (CMO) mice, when severe multifocal osteomyelitis develops (Cassel et al., 2014; Ferguson et al., 2006; Lukens et al., 2014a). Compared with baseline levels of Alcian Blue-stained CTMCs in WT tail tissue sections, we observed a trend towards increased CTMCs in CMO tail tissues (Fig. 1A,B). To test for mast cell activation and mediator release, we collected mouse plasma at 5 months of age and measured the levels of mouse mast cell protease-1 (MCPT1) by ELISA. MCPT1 levels were significantly elevated in plasma from CMO mice, as compared with WT animals (Fig. 1C). These results suggest that CTMCs are present within inflamed tissues of CMO mice and that mast cell mediators are produced in this autoinflammatory disease model.

To test the contribution of CTMCs to the development of CMO disease, we employed a transgenic model that results in constitutive ablation of CTMCs as a result of diptheria toxin-α expression in mature CTMCs (*Mcpt5-Cre:Rosa26-Stop*^fl/fl^*-DTa*) (Dudeck et al., 2011). Both transgenes were crossed with CMO mice (Ferguson et al., 2006), and backcrossed more than nine generations to produce CTMC-deficient CMO animals (hereafter referred to as CMO/MC^–^) on a uniform Balb/c background. We compared CTMC densities in ear skin tissue sections from WT, CMO and CMO/MC^–^ mice using Alcian Blue staining. Compared with WT, we observed elevated CTMC density in CMO ear skin lesions (Fig. 1D). CMO/MC^–^ ear skin was largely devoid of CTMCs (Fig. 1D), which also correlated with reduced inflammation and edema (Fig. S1). These results suggest a potential role for CTMC activation in CMO disease and demonstrate the efficacy of mast cell ablation in the CMO/MC^–^ model.

To determine the role of CTMCs in CMO disease onset, cohorts of age-matched CMO and CMO/MC^–^ mice were monitored for early symptoms of CMO, including tail kinks or deformities in hind paws. The CMO/MC^–^ cohort showed a significant delay in disease onset compared with CMO mice, whereas WT mice showed no disease (Fig. 1E,F). Overall, these results implicate mast cells in promotion of inflammation in the CMO model.

### CTMC deficiency protects against bone damage in CMO mice

**Fig. 2.**
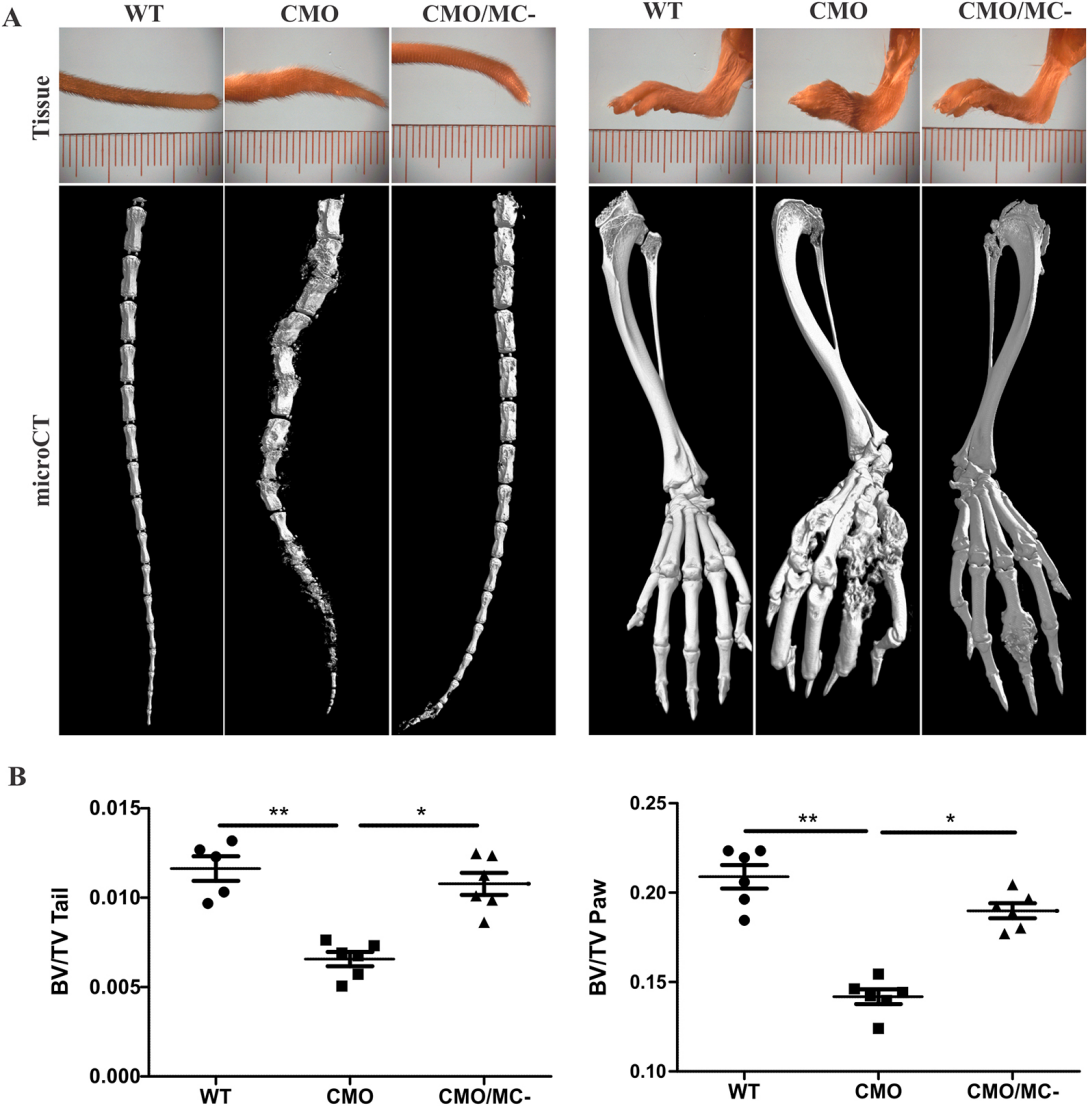
**CTMCs promote bone lesion severity in CMO mice.** (A) Representative images of tails and hind paw tissues, along with µCT images of underlying bone lesions in male 5-month-old WT, CMO and CMO/MC^–^ mice. (B) Quantification of bone volume to total volume ratios for each stack of µCT images of tails and hind paws for each cohort (*n*=6 per group). *P<0.05, **P<0.01.

To test the role of CTMCs in CMO disease progression, tail and paw tissues were collected from cohorts of male WT, CMO and CMO/MC^–^ mice at 5 months of age. Analysis of these tissues and microcomputed tomography (µCT) scans revealed fewer deformities and bone lesions within the tails and paws of CMO/MC^–^ mice compared with CMO mice (Fig. 2A). Quantification of bone density from the µCT scans of six mice from each group revealed that the CMO/MC^–^ cohort was significantly protected from the bone loss observed in CMO mice (Fig. 2B). This also correlated with fewer TRAP-positive osteoclasts in CMO/MC^–^ compared with CMO tail tissue sections (Fig. S2). These results indicate that CTMCs promote bone inflammation and the resulting damage by osteoclasts that are hallmarks of this autoinflammatory disease.

### CTMCs promote IL-1β production and immune cell infiltration in CMO mice

**Fig. 3.**
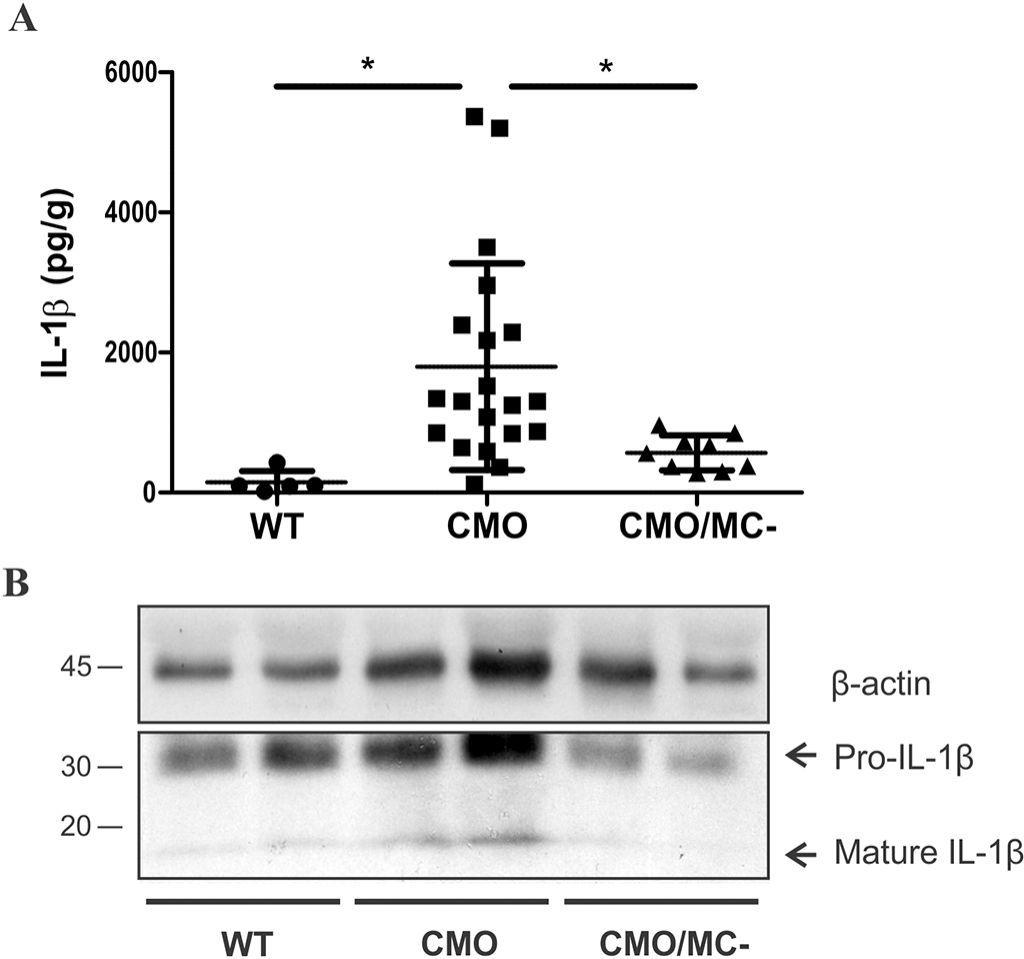
**CTMC deficiency impairs IL-1β production in CMO disease tissue.** (A) Levels of IL-1β detected by ELISA using tail tissue homogenates from 5-month-old male WT (*n*=5), CMO (*n*=18), and CMO/MC^–^ mice (*n*=9; *P<0.05). (B) Representative immunoblot detection of pro-IL-1β and mature IL-1β in homogenates of tail tissues from WT, CMO and CMO/MC^–^ male mice at 5 months of age. The positions of molecular weight markers (left) and pro-IL-1β and mature IL-1β (right) are shown. The β-actin immunoblot was used as a loading control.

Previous studies of CMO mice revealed a crucial role for elevated IL-1β production and signaling in this model (Cassel et al., 2014; Lukens et al., 2014a). To elucidate how CTMCs contribute to IL-1β production, we prepared tail tissue homogenates from 5-month-old WT, CMO and CMO/MC^–^ mice and measured IL-1β levels by ELISA. Consistent with previous studies (Cassel et al., 2014; Lukens et al., 2014a), CMO tissues had significantly higher IL-1β levels compared with WT mice, but these levels were largely normalized in CMO/MC^–^ mice (Fig. 3A). Using immunoblot assays to separate pro-IL-1β and mature IL-1β, we showed that CMO/MC^–^ tail tissues had less of the mature form of IL-1β (Fig. 3B). No differences in serum levels of IL-1β were observed in these mice (data not shown). These results suggest that CTMCs either contribute to IL-1β production in the diseased tissues of CMO mice or promote recruitment of other immune cells that produce IL-1β in this model.

**Fig. 4.**
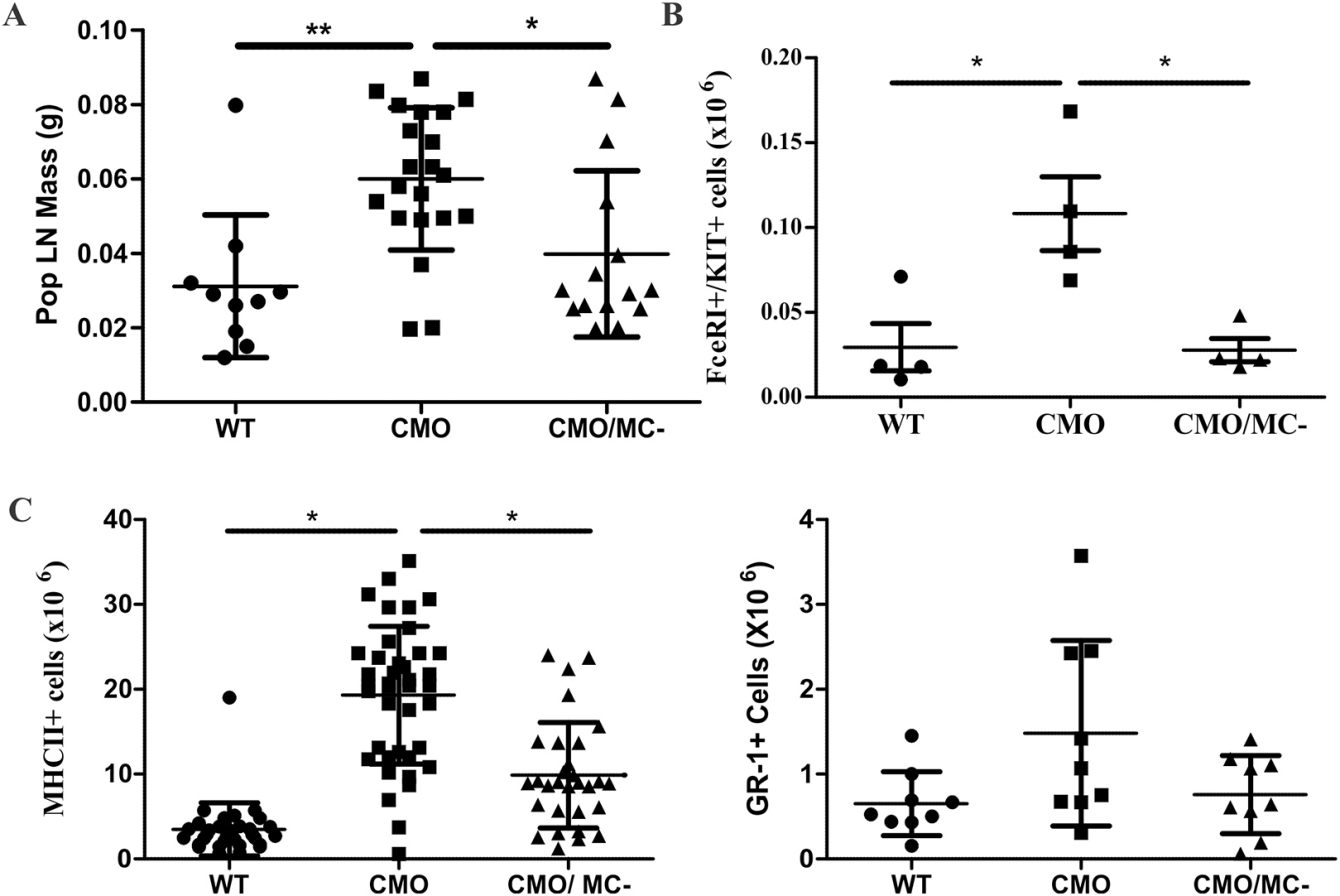
**Impaired immune cell recruitment to popliteal lymph nodes in CMO/MC^–^ mice.** (A) Mean mass of popliteal lymph nodes for WT, CMO, and CMO/MC^–^ male mice (*n*=10-20/group). (B) Absolute numbers of mast cells (FcεRI^+^/KIT^+^) in popliteal lymph nodes from WT, CMO and CMO/MC^–^ male mice based on flow cytometry (*n*=4/group). (C) Absolute numbers of MHCII^+^ and GR-1+ immune cells in popliteal lymph nodes from WT, CMO and CMO/MC^–^ male mice based on flow cytometry (*n*=9-21/group; results pooled from repeated experiments). *P<0.05, **P<0.01.

Prior studies correlated the severity of CMO with the size of popliteal lymph nodes that ‘drain’ the tail and hind paws (Chitu et al., 2009; Lukens et al., 2014a). Indeed, popliteal lymph node mass was elevated in CMO mice compared with WT animals, but this expansion was reduced in CMO/MC^–^ mice (Fig. 4A). Flow cytometric analyses of immune cell markers in lymph nodes revealed that CMO mice had significantly elevated numbers of FcεRI^+^/KIT^+^ mast cells, MHCII^+^-activated immune cells and a trend towards increased Ly6G^+^/Ly6C^+^ (GR-1) myeloid cells in lymph nodes compared with either WT or CMO/MC^–^ mice (Fig. 4B). Together, these results support increased mast cell density in CMO disease tissues and a role of CTMCs in the recruitment of immune cells in the inflamed paw and tail tissues of CMO mice.

### Elevated cytokine production in cultured CMO mast cells

**Fig. 5.**
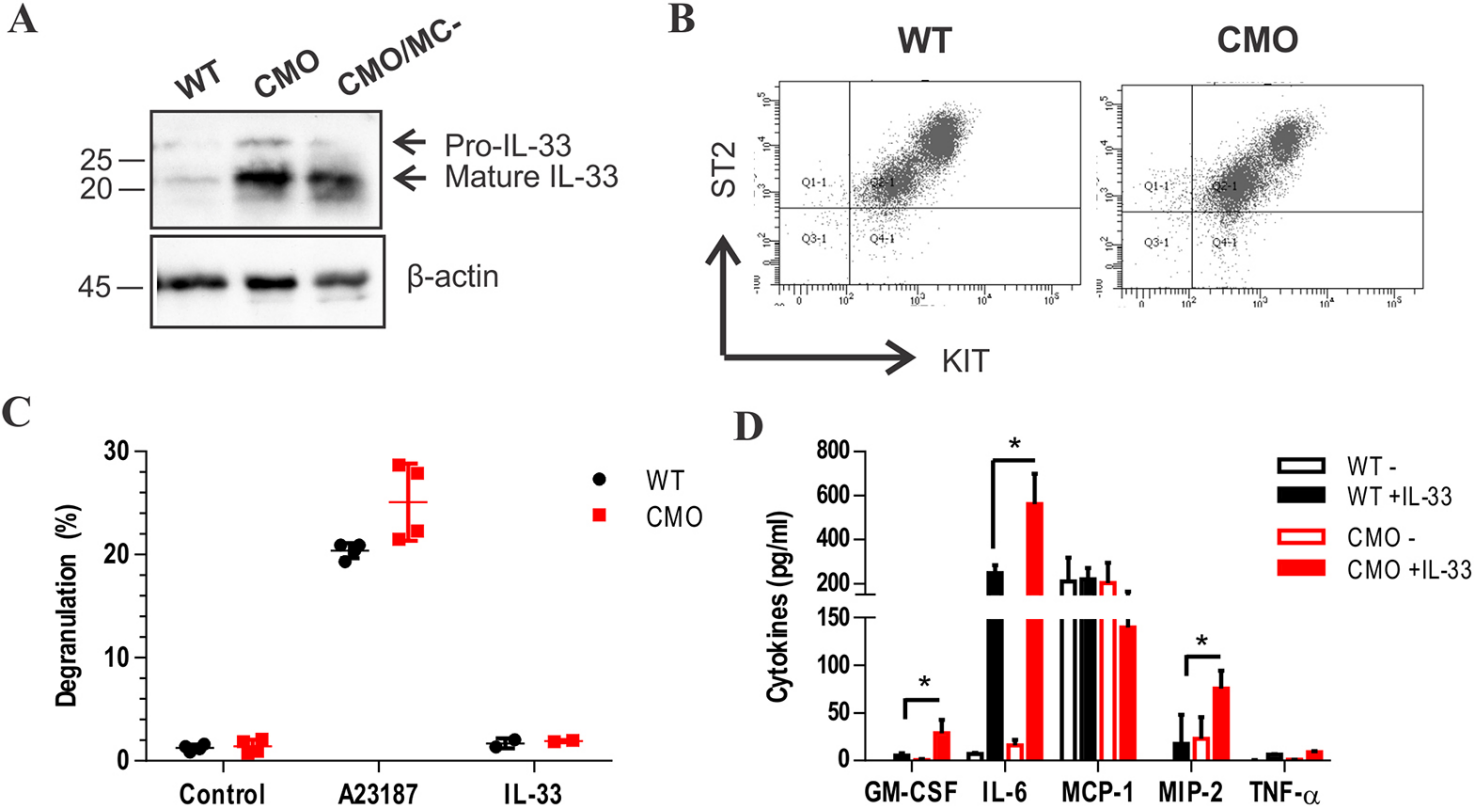
**Increased cytokine production by CMO BMMCs treated with IL-33.** (A) Representative immunoblot detection of pro-IL-33 and mature IL-33 in homogenates of tail tissues from WT, CMO and CMO/MC^–^ male mice at 5 months of age. The positions of molecular weight markers (left) and pro-IL-33 and mature IL-33 (right) are shown. The β-actin immunoblot was used as a loading control. (B) Representative flow cytometry histograms of ST2 and KIT expression in WT and CMO BMMCs. (C) Mast cell degranulation in WT and CMO BMMCs following treatment with or without calcium ionophore (A23187) or IL-33 for 15 min (*n*=4/genotype). (D) Cytokine production for WT and CMO BMMCs following treatment with or without IL-33 for 18 h (*n*=3/genotype). *P<0.05.

Next, we examined potential mast cell activation pathways and mechanisms to account for mast cell-related defects in CMO mice. We considered the alarmin IL-33 as a candidate mast cell activator in the CMO model because of its involvement in sterile inflammation and high expression of the IL-33 receptor ST2 in mast cells. We analyzed IL-33 levels and processing in tail tissues from 5-month-old WT, CMO and CMO/MC^–^ mice. IL-33 levels were higher in CMO and CMO/MC^–^ tissues compared with WT mice, consistent with increased levels with inflammation and disease (Fig. 5A). It is worth noting that the mature form of IL-33 was most abundant in both CMO and CMO/MC^–^ tissues, suggesting processing by non-CTMC proteases in this model.

To test whether mast cells from CMO mice have cell autonomous defects in their activation state, we prepared bone marrow-derived mast cell (BMMC) cultures from WT and CMO mice. After 4 weeks in culture in medium supplemented with IL-3 and SCF, we observed similar levels of KIT and ST2 expression between genotypes (Fig. 5B). To assess degranulation, we measured β-hexosaminidase release and observed no significant difference in degranulation in CMO compared with WT BMMCs that were either untreated or treated with IL-33 (Fig. 5C). Addition of the calcium ionophore A23187 triggered degranulation in both genotypes, to a similar extent (Fig. 5C). We further tested *de novo* cytokine production in WT and CMO BMMCs treated with or without IL-33 for 18 h. Several cytokines or chemokines (GM-CSF, IL-6, MIP-2) were produced at higher levels in CMO compared with WT BMMCs in response to IL-33 treatment (Fig. 5D). In contrast, the constitutively produced chemokine MCP-1 was produced at similar levels in WT and CMO BMMCs (Fig. 5D). Together, these results provide evidence of a proinflammatory phenotype of CMO mast cells when triggered by the alarmin IL-33.

### Mast cell activation in CRMO patient samples

**Fig. 6.**
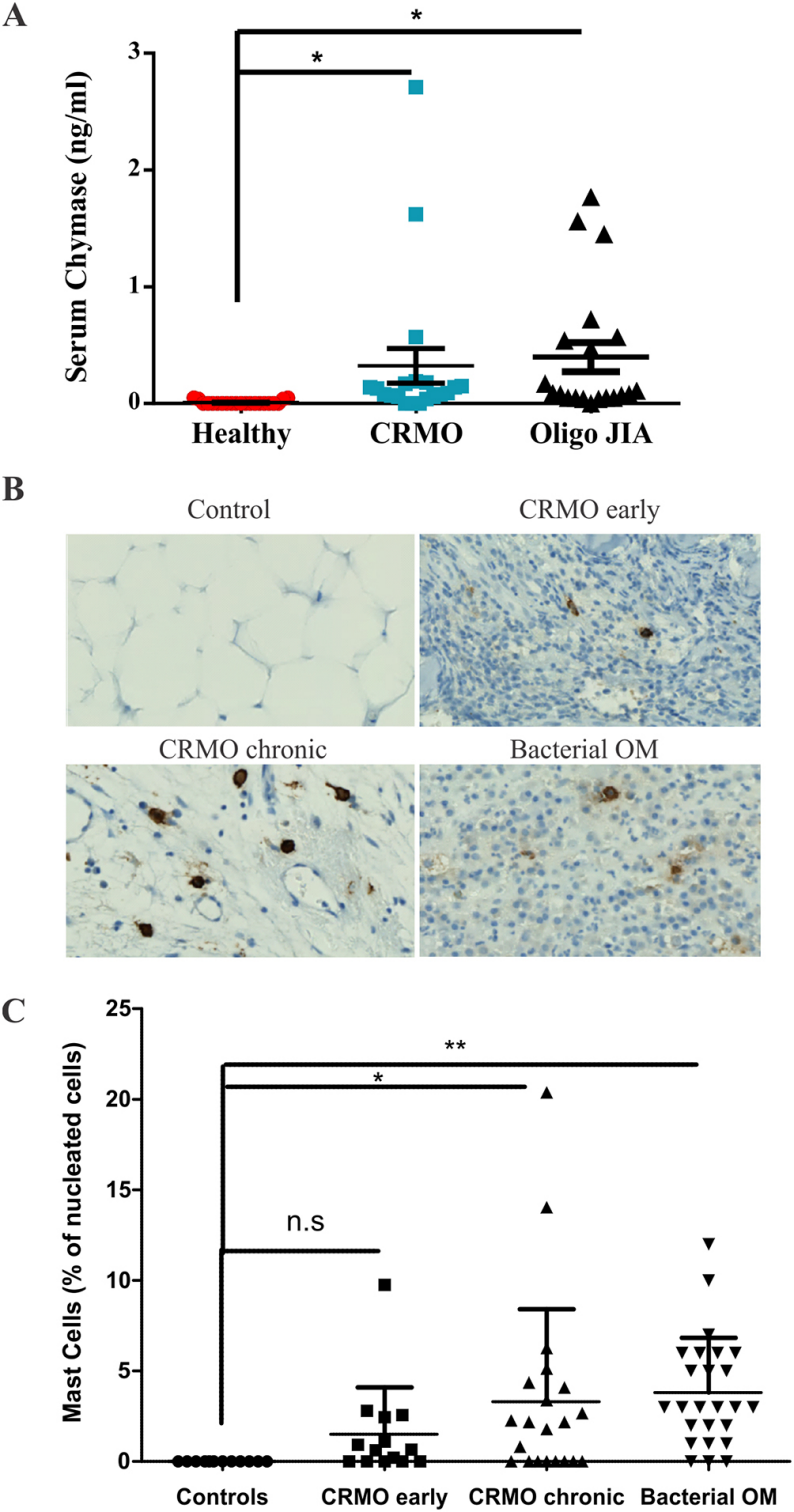
**Detection of mast cells and mast cell mediators in CRMO patient samples.** (A) Serum samples from human patients with CRMO (*n*=20), oligoarticular JIA patients (*n*=20) or healthy controls (*n*=21) were tested for the levels of mast cell chymase by ELISA. Dot plot depicts individual values with median and interquartile range overlaid. (B) Representative images of tryptase staining of bone from healthy controls, early and chronic CRMO patients and infectious osteomyelitis patients. (C) Percentage of tryptase-positive mast cells relative to total nucleated cells in the field of view for bone sections from healthy controls, early and chronic lesions from CRMO patients, and bacterial OM patients. *P<0.05, **P<0.01.

To test the potential involvement of mast cells in human CRMO, we screened sera from a previously reported cohort of treatment-naïve, newly diagnosed CRMO patients, oligoarticular juvenile arthritis (Oligo JIA) patients, and healthy controls (Hofmann et al., 2016a). We tested for mast cell chymase by ELISA and detected very low levels of chymase in 4 of 21 healthy controls, whereas the vast majority of CRMO patients (17 of 20) exhibited detectable serum chymase levels (Fig. 6A). No patients in these cohorts had reported allergies. Of note, a comparable increase in serum chymase levels was also observed in Oligo JIA patients (Fig. 6A), which is consistent with a recent study implicating mast cells in arthritis disease models (Schubert et al., 2015).

To assess mast cell infiltration to inflamed bone tissue, we performed immunohistochemistry staining of tryptase-positive mast cells in tissue sections from bone biopsies taken from healthy controls (osteotomies), CRMO patients, and bacterial osteomyelitis (OM) patients. Although no mast cells were detected in bone biopsies from healthy individuals, we detected mast cells in CRMO lesions, including early CRMO lesions marked by innate immune infiltrates (Fig. 6B,C). Particularly high mast cell counts were detected in chronic CRMO lesions marked by coexisting infiltrates of innate immune cells and lymphocytes (Fig. 6B,C). Mast cell counts were also increased in bacterial OM bone biopsies compared with controls (Fig. 6B,C). Together, these results provide evidence of mast cell involvement in autoinflammation in the bone of patients with CRMO and related disorders.

## DISCUSSION

**Fig. 7.**
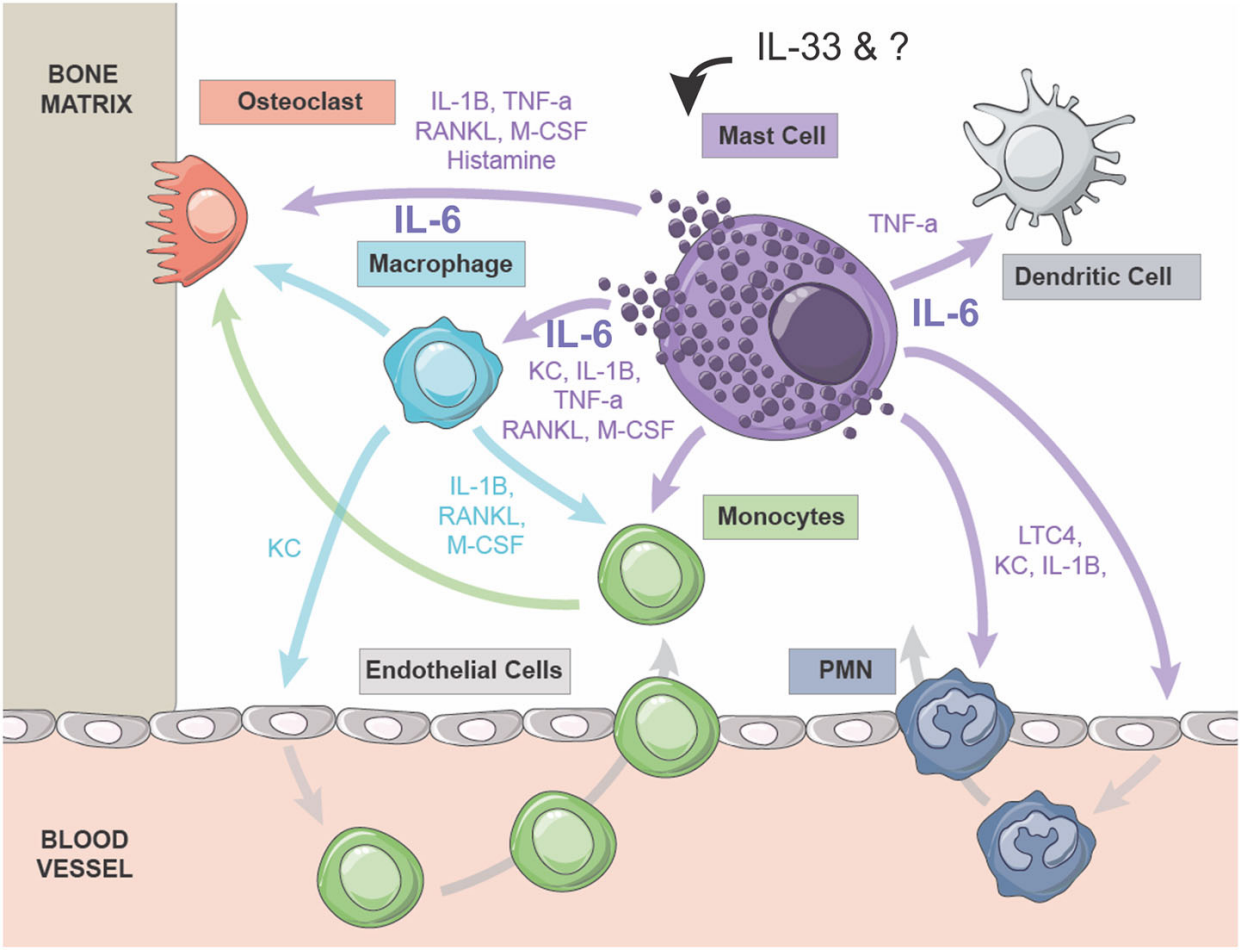
**Hypothetical model of the potential crosstalk between mast cell mediators and cell types implicated in autoinflammation.** Model depicts the effects of mast cell activation by alarmin IL-33 or other triggers, and the effects of subsequent mediator release on the recruitment and activation of innate immune cells and osteoclasts implicated in promoting IL-1β-driven sterile bone inflammation. The effects of IL-6 and other mast cell mediators on these cell types might promote inflammation in this model (see text for details). KC, keratinocyte chemoattractant (CXCL1); LTC4, leukotriene C4; M-CSF, macrophage colony stimulating factor; PMN, polymorphonuclear leukocytes; RANKL, receptor activator of nuclear factor kappa-B ligand; TNF-a, tumor necrosis factor alpha.

Studies in CMO mice and related mouse models have provided insights into the pathophysiology of human CRMO, a rare autoinflammatory disease. This includes the identification of a skewed microbiome, increased IL-1β production and aberrantly activated innate immune cells (Cassel et al., 2014; Chitu et al., 2009, 2012; Lukens et al., 2014a,b). In the study presented here, we show that mast cells accumulate in CMO lesions and promote the accumulation of bone inflammation and lesions. By crossing CMO mice with CTMC-deficient animals (Dudeck et al., 2011), we provide evidence that CTMCs promote CMO disease onset and severity. To address cell autonomous mast cell defects in the CMO model, we show that CMO BMMCs produce elevated levels of inflammatory cytokines in response to treatment with the alarmin IL-33, which is elevated in CMO disease tissues. We also translate these studies to human CRMO by providing evidence of mast cell infiltrates in bone biopsies from CRMO patients, and elevated levels of mast cell chymase in the serum of CRMO patients at diagnosis. Together, these findings implicate mast cells in promoting bone inflammation in CMO mice and suggest a role for mast cells in the pathophysiology of CRMO in humans. Our model in Fig. 7 depicts several candidate mediators from mast cells, including IL-6, that promote recruitment and activation of the innate immune cells and osteoclasts that trigger autoinflammation.

A recent study provides evidence of functional crosstalk between mast cells and osteoclasts, with mast cell degranulation promoting osteoclast differentiation and activation in bone fracture and osteoporosis models (Kroner et al., 2017). Mast cell-derived TNF-α has also been reported to promote bone loss in periodontitis induced by oral cavity infection with Porphyromonas gingivalis (Malcolm et al., 2016). Our study also suggests that mast cell activation promotes osteoclast expansion and bone loss in the CMO model. It is worth noting that osteoclast differentiation from immature myeloid cells has been reported in arthritis (Zhang et al., 2015a), and it will be worth testing whether mast cells contribute to this process in CMO/CRMO. Our findings that CMO mast cells release more IL-6 could explain increased osteoclast numbers and bone loss based on the role of IL-6 in collagen-induced arthritis models (Tanaka et al., 2014). Mast cell-derived IL-6 has also been implicated in mobilization of dendritic cell subsets linked to inflammation (Dawicki et al., 2010). It is worth noting that mast cells might participate in a bone reparation or remodeling pathway that is co-opted in the CMO model and that mast cells could have both positive and negative effects on bone inflammation overall. Future studies are warranted in order to fully dissect the potential crosstalk between mast cells, dendritic cells, osteoclasts and other relevant cell types in CRMO and related diseases.

Because CMO mice are protected from disease when crossed onto either IL-1R-null or IL-1β-null backgrounds (Cassel et al., 2014; Lukens et al., 2014a), mast cells could be an important source of IL-1β in this disease model. Alternatively, the reduced levels of IL-1β in CMO/MC^–^ mice might be a result of impaired recruitment of IL-1β-producing immune cells. Indeed, draining lymph nodes from CMO/MC^–^ mice contained fewer innate immune cells compared with CMO mice. Mast cell proteases released in response to activation could also enhance cleavage of pro-IL-1β in inflamed tissues, based on studies in other models (Caughey, 2007; Dong et al., 2012). Interestingly, chymase is elevated in serum from CMO mice and CRMO patients at diagnosis. Thus, chymase could indeed participate in CMO and CRMO pathophysiology and is worthy of further functional studies.

Previous studies suggested that neutrophils in CMO produce aberrant levels of IL-1β in an inflammasome-independent manner, which might be responsible for driving the CMO disease pathophysiology (Cassel et al., 2014; Lukens et al., 2014b). Indeed, depletion of neutrophils using a Ly6G antibody protected CMO mice from bone lesion development (Lukens et al., 2014b). It is worth noting that granulocytic myeloid-derived suppressor cells (G-MDSCs) express both Ly6C and Ly6G markers (Ly6C^lo^/Ly6G^hi^), and are recruited to bone as the main osteoclast precursor population (Sawant and Ponnazhagan, 2013; Zhang et al., 2015a). These cells then differentiate into osteoclasts in chronic inflammatory disorders such as arthritis and bone cancer (Fujii et al., 2013; Sawant and Ponnazhagan, 2013; Zhang et al., 2015a,b). Thus, ‘neutrophil’ depletion with inactivating anti-Ly6G antibodies in CMO mice (Lukens et al., 2014b) might have also depleted osteoclast precursor G-MDSCs, which would lead to fewer osteoclasts and reduced bone resorption. Interestingly, MDSC proliferation has been reported to be mast cell dependent (Hegde et al., 2015). Further studies using more selective tools to deplete and phenotype the inflammatory cells in this model are needed to define CMO disease pathophysiology better.

The identity of potential triggers of CMO disease and restrictions of the phenotypes to certain tissues remains poorly defined. A previous study identified a skewed microbiome in CMO mice that leads to priming of neutrophils to express IL-1β (Lukens et al., 2014b). Based on the selective loss of CTMCs in our model, with no effect on mucosal mast cells (Dudeck et al., 2011; Sharma et al., 2012), there could be mast cell involvement at the interface with a skewed microbiome but we have not yet tested this. However, future studies using Cpa3^Cre^ mice, lacking both mucosal and connective tissue mast cells (Feyerabend et al., 2011), would provide an improved model to test the overall contribution of mast cells to this disease. Our findings in this study also identified elevated levels of mature IL-33 in the tail tissues of diseased CMO and CMO/MC^–^ mice, and showed that IL-33 might trigger aberrant mast cell activation in this model. It is worth noting that transgenic mice with IL-33 overexpression in the myeloid compartment develop severe neutrophil-driven inflammation in several tissues, including the paws (Talabot-Ayer et al., 2015). As in CMO mice (Chitu et al., 2009), elevated proinflammatory cytokines such as IL-1β and IL-6 have been linked to IL-33-driven sterile inflammation (Talabot-Ayer et al., 2015). It is also noteworthy that levels of IL-33 and soluble ST2 were elevated in serum of patients with the autoinflammatory Behcet's disease (Kim et al., 2013). Further studies of the IL-33–ST2 axis as a biomarker or therapeutic target in CMO/CRMO are certainly warranted.

To translate findings from our study of the involvement of mast cells in murine CMO to CRMO in humans, we stained bone samples from CRMO patients. Our results demonstrated increased mast cell populations in CRMO patients. Profiling of CRMO serum biomarkers highlighted a number of cytokines and chemokines that were elevated at diagnosis (Hofmann et al., 2016a), several of which are known to be released by activated human mast cells (Dawicki et al., 2010; Oldford et al., 2010; Sugitharini et al., 2016). Our detection of elevated levels of mast cell chymase in sera from most CRMO patients provides new evidence of mast cell activation in this disease. This was also true for Oligo JIA patient sera, suggesting a common immune mechanism involving mast cells in these diseases. Of note, chymase can directly promote IL-1β production (Mizutani et al., 1991), and other mast cell mediators might also contribute to IL-1β-driven disease in CRMO patients (Hofmann et al., 2015, 2016a). This is also consistent with our findings of elevated mast cell density in bone tissue sections from CRMO and bacterial OM patients compared with healthy controls. A parallel study of other immune cell types was recently reported for this panel of patient samples, with significant increases in CD14^+^ monocytes, CD15^+^ neutrophils, lymphocytes and plasma cells (Brandt et al., 2018). Future studies are needed to examine the potential mast cell interactions with these immune cells in CRMO and bacterial OM disease samples.

In conclusion, we demonstrate a role for mast cells in the progression of autoinflammatory bone disease using mouse models and human CRMO patient samples. We show that aberrant mast cell activation enhances the cycle of IL-1β-driven sterile inflammation that triggers bone loss in this disease (Fig. 7). Indeed, there is growing evidence of mast cell involvement in autoinflammation in a variety of models and diseases (Bonnekoh et al., 2018). This study provides the rationale to study the effects of mast cell-targeted therapies in CNO and related autoinflammatory bone diseases.

## MATERIALS AND METHODS

### Mouse studies

The constitutive CTMC ablation model (*Mcpt5-Cre:Rosa-Stop*^fl/fl^*-Dta*) (Dudeck et al., 2011) was crossed for 10 generations with *Pstpip2*^cmo/cmo^ (CMO) mice (Chitu et al., 2012) to achieve a uniform BALB/cAnPt background for all (CMO/MC^–^) mice. All animals were housed at Queen's University Animal Care Services under specific pathogen-free conditions. All procedures were approved by the Queen's University Animal Care committee in accordance with Canadian Council on Animal Care guidelines.

### Disease scoring, pharmacological treatments and preparation of tissue homogenates

Wild-type (WT), CMO and CMO/MC^–^ mice were monitored for the initial appearance of a tail kink or paw deformity to record the date of disease onset. For tissue cytokine measurements, equal weights of distal tail segments and hind paws were snap-frozen, homogenized and assayed for IL-1β (e-bioscience) using ELISA. To distinguish levels of pro- and mature forms of IL-1β and IL-33, some homogenates were analyzed by immunoblot using mouse anti-IL-1β (3A6, Cell Signaling Technology) and goat anti-IL-33 (R&D Systems); anti-β-actin (Santa Cruz Biotechnology) served as a loading control.

### Lymph node immune profiling

Popliteal lymph nodes from each mouse were collected, weighed and manually homogenized in PBS-EDTA solution. Cells were counted, resuspended in PBS containing sodium azide (0.1%) and BSA (0.5%), and stained with FITC anti-CD117 (c-KIT, BioLegend), PE anti-FcεRI (BioLegend), FITC anti-I-Ek/RT1-D (MHC II, BioLegend), FITC anti-Ly6C (BD Biosciences) or PE anti-Ly6G (BD Biosciences). Samples were analyzed using a cytomics Fc500 (Beckman Coulter), and FlowJo software (Tree Star), with gating set to exclude dead cells (low forward and side scatter); isotype control antibodies were used to set threshold values.

### Bone marrow-derived mast cell cultures and treatments

Bone marrow-derived mast cell (BMMC) cultures were established from femoral bone marrow cells in the presence of IL-3 and stem cell factor (SCF) as described previously (McPherson et al., 2009; Samayawardhena et al., 2007; Udell et al., 2006). BMMC maturation and ST2 expression were assessed by staining with KIT (FITC anti-KIT, BioLegend) and ST2 (PE-Cy7 anti-ST2, BioLegend), and analyzed using a FACS Aria III (Beckman Coulter). Gating was set to exclude dead cells (low forward and side scatter); isotype controls were used to set threshold values. Degranulation of BMMCs was measured upon treatment with or without calcium ionophore A23187 (1 µM), or IL-33 (10 ng/ml; Peprotech) for 15 min using a β-hexosaminidase release assay. To measure cytokine production, mature BMMCs were treated with or without IL-33 (10 ng/ml) for 18 h. Conditioned medium was collected for cytokine detection using a multiplex panel of mouse cytokines (mouse high-sensitivity 18-plex assay, Eve Technologies Inc.).

### Microcomputed tomography

Tail and hind paws harvested from male mice at 5 months of age were analyzed in a Skyscan 1172 high-resolution µCT scanner (Bruker, Milton, Canada). With the X-ray source at 100 kVp and 100 µA, and the use of a 0.5 mm aluminium filter, each specimen was rotated 360° around the vertical axis to generate 1200 views in 5 h. The image projections were reconstructed into digital cross-sections using the Feldkamp algorithm (Feldkamp et al., 1984) for cone beam CT. The resulting 3D data block contained 2000×1000×1000 voxels of 13.4 µm, resampled to match the resolution (28 µm) and data size of the 3D segmented atlas. Down-sampling of the 3D images was performed because the 2× decrease in voxel size allowed 8× reductions in the amount of RAM used and image processing times. Volume fraction data (BV/TV) for serial sections were analyzed with ImageJ software plugin BoneJ (Doube et al., 2010).

### Human CRMO patient serum and bone biopsy analyses

The cohort of CRMO patient serum samples taken at initial diagnosis was described previously (Hofmann et al., 2016a). Chymase levels were measured by ELISA (Cusabio) at University Hospital Carl Custav Carus (Dresden, Germany) in sera from 20 CRMO patients and 21 matched controls. Patients and controls had no records of allergies in their patient records, and all patients and/or their legal guardians gave written informed consent (Ethics Committees at University of Würzburg and University of Technology Dresden) in accordance with the Declaration of Helsinki principles (Hofmann et al., 2016a). Formalin-fixed, decalcified and paraffin-embedded bone biopsy specimens from healthy control osteotomies (*n*=2) and patients with bacterial OM (*n*=3), early CRMO lesions (*n*=4) or chronic phase CRMO lesions (*n*=4) (Brandt et al., 2018) were stained with an anti-human-tryptase antibody (Abcam, ab2378, dilution: 1:500) using standard techniques. Nuclei were stained using hematoxylin and Bluing reagent on the BenchMarkXT staining system (Ventana Medical Systems/Roche). Slides were scanned on the AxioScan Z1 (Zeiss, Germany) and quantified using Zen Blue software (Zeiss, Germany) in 15-25 fields of view (32,188.774 µm^2^).

### Statistical analysis

To compare the three genotypes, we used Kruskal–Wallis nonparametric testing with multiple comparison testing (GraphPad Prism Software, La Jolla, California).

## Supplementary Material

Supplementary information
