# Supplementary material for: Mast cells enhance sterile inflammation in chronic nonbacterial osteomyelitis
Source: Dis Model Mech. 2019 Aug 20;12(8):dmm040097. doi: 10.1242/dmm.040097 (PMC6737947; doi:10.1242/dmm.040097)
Supplement: Supplementary information [file dmm-12-040097-s1.pdf]

Appendix A. Supplementary Figures

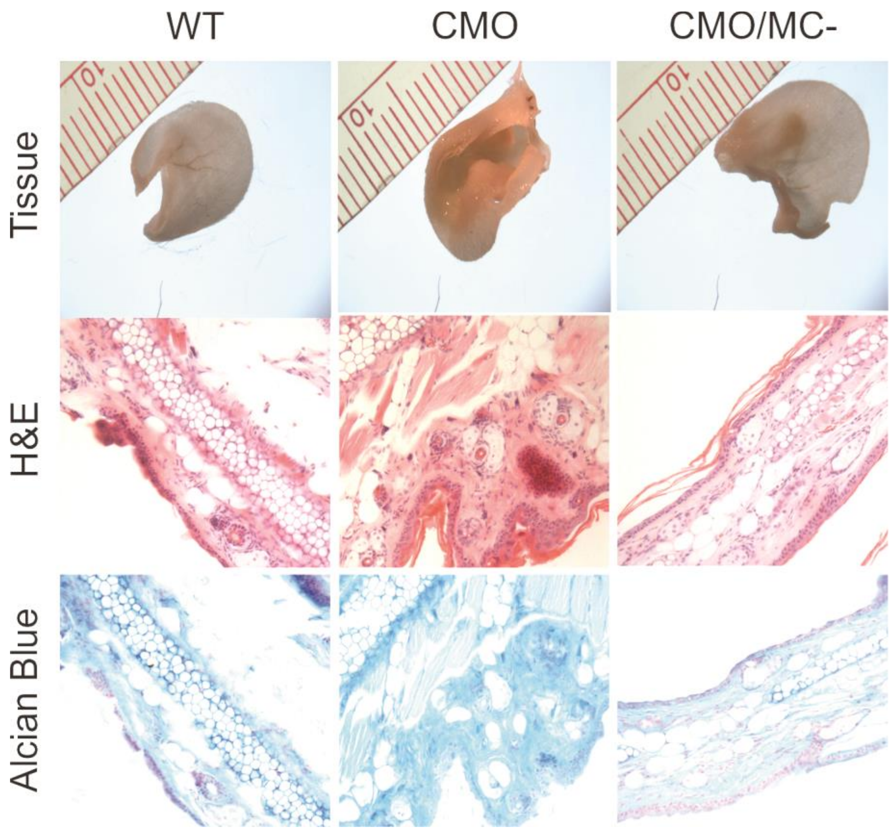

Fig. S1. Connective tissue mast cell density increases in CMO skin lesions and efficacy of mast cell ablation. Representative images of 5 month old WT, CMO and CMO/MC- male mouse tissues, including ears (upper panels) and ear tissue sections (lower panels) stained with H&E or Alcian blue.

Disease Models & Mechanisms • Supplementary information

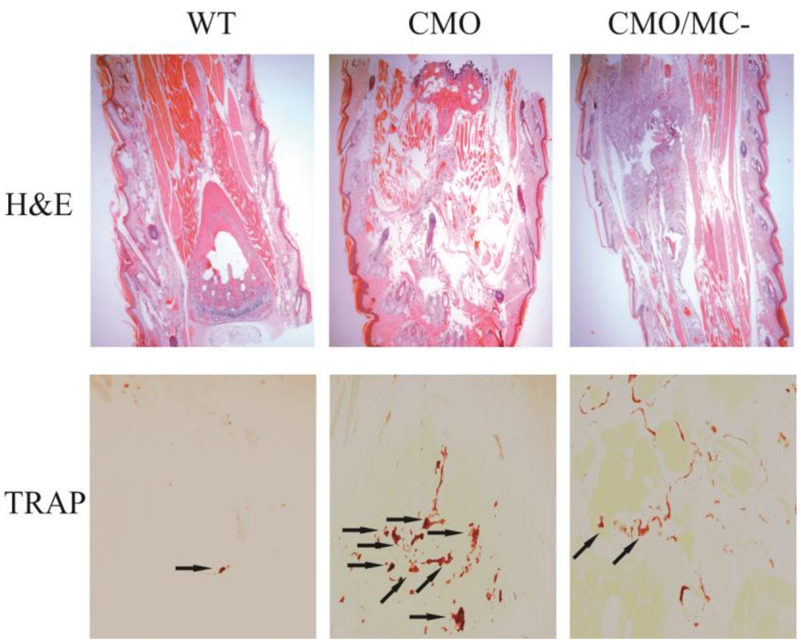

Fig. S2. Connective tissue mast cells promote osteoclast accumulation in CMO mice. Representative histological images of decalcified tail tissue sections stained with H&E (upper panels) and TRAP (lower panels) for male WT, CMO and CMO/MC- mice at 5 months of age (arrows indicate TRAP+ osteoclasts).

Disease Models & Mechanisms • Supplementary information
